# Skilled birth attendance in Sierra Leone, Niger, and Mali: analysis of demographic and health surveys

**DOI:** 10.1186/s12889-020-8258-z

**Published:** 2020-02-03

**Authors:** Edward Kwabena Ameyaw, Kwamena Sekyi Dickson

**Affiliations:** 1 The Australian Centre for Public and Population Health Research, Faculty of Health, University of Technology Sydney, Sydney, NSW Australia; 2 Department of Population and Health, College of Humanities and Legal Studies, University of Cape Coast, Cape Coast, Ghana

**Keywords:** Skilled birth attendance, Women, Mali, Niger, Sierra Leone

## Abstract

**Background:**

Skilled birth attendance (SBA) is a key strategy for averting maternal mortality ratio (MMR). The lifetime risk of maternal death is high in countries with low SBA. With the presence of a skilled birth attendant, the possibility of death owing to intrapartum-related complications or stillbirth can be reduced by 20%.

**Methods:**

Using data from the most recent Demographic and Health Surveys, we investigated the prevalence of skilled birth attendance, variations, and associated factors. The sample was drawn from women aged 15–49 who were surveyed in these countries as part of the Demographic and Health Survey (DHS) program. With multivariate logistic regression, we explored the socio-demographic factors that predict women’s likelihood of seeking skilled birth attendance or otherwise.

**Results:**

Less than half of the women in Niger, Sierra Leone, and Mali obtained skilled birth attendance, with the worst case occurring in Niger (32.6%). Women in rural areas have less likelihood of obtaining skilled birth attendance (OR 0.21; 95% CI 0.16–0.28), as compared to women in urban locations. Highly educated women (OR 2.50; 95% CI 0.72–8.69), those who had subscribed to health insurance (OR 1.39; 95% CI 0.88–2.20), those who obtain four or more antenatal care visits (OR 1.63; 95% CI 1.43–1.86), and women who watch television at least once a week (OR 2.33; 95% CI 1.88–2.88) are more probable to seek SBA.

**Conclusion:**

Interventions to increase SBA rates in these countries need to be reassessed to focus on the rural-urban disparity in healthcare, female education, and ANC attendance.

## Background

Skilled birth attendance (SBA) is a key strategy for averting maternal mortality ratio (MMR) [1]. The lifetime risk of maternal death is high in countries with low SBA [2]. With the presence of a skilled birth attendant, the possibility of death owing to intrapartum-related complications or stillbirth can be reduced by 20% [3]. Elevating SBA rates for women can, therefore, contribute substantially toward the realization of the Sustainable Development Goal (SDG) 3. SDG three inter alia envisages the reduction of maternal mortality to 70 per 100, 000 maternal mortality by 2030 [4]. To achieve this, SBA, which is delivery assisted by a skilled birth attendant, has been recognized as a protective mechanism for saving maternal and newborn lives [5–7]. A skilled birth attendant is “an accredited health professional such as a midwife, doctor, or nurse who has been educated and trained to proficiency in the skills needed to manage normal (uncomplicated) pregnancies, childbirth, and the immediate postnatal period, and in the identification, management, and referral of complications in women and newborns” [8].

Some evidence indicates that only 17% of women attain SBA in Niger, with most of them delivering at home with TBA’s assistance [9, 10]. The head of the United Nations Children’s Fund (UNICEF) Niger’s Maternal Health Programme stated, “Even if there were more doctors and hospitals, women would most likely not go. Due to culture, they prefer to give birth at home” [10]. This implies the absence of skilled birth attendances for most women during labour. Building more health facilities has been suggested but, as purported by the framework for evaluation of quality care in maternity services, availability of health facilities does not commensurate with utilisation [11]. It has been reported that 14,000 women die from pregnancy-related causes, with several others experiencing disabilities, infections, and varying degrees of injuries [10]. Almost 80% of Niger women marry by 18 and 40% before age 15. Childbirth at these tender ages partly accounts for the high MMR. A woman’s lifetime risk of dying as a result of childbirth or pregnancy complication is one in seven in Niger [12].

The situation in Mali does not vary from what is happening in Niger because 1 in 2 young women between 20 and 24 years give birth by 18 years, as revealed by the Demographic and Health Survey [13]. The maternal health situation in Mali is one of the poorest in sub-Saharan Africa in spite of the proliferation of maternal and child health driven interventions such as the USAID’s Maternal and Child Survival Program (MCSP) [14]. Vast rural-urban SBA disparity exists – 51 and 92% SBA in rural and urban settings respectively [13]. Exhibition of disrespectful and hostile behaviour by some skilled birth attendants has been documented as a possible disincentive to SBA [15]. Sierra Leone is another country in sub-Saharan Africa with similar features.

In 2016, most maternal deaths in Sierra Leone were induced by postpartum hemorrhage, a situation which could be handled by a competent skilled birth attendant under normal circumstance [16]. Pregnant women have an approximate lifetime risk of maternal mortality of 1 in 17 in Sierra Leone [17]. To improve the situation, the government introduced the Free Health Care Initiative (FHCI) in 2010 to exempt women from costs associated with maternal healthcare services [18]. However, not much has been achieved, as the country still records the highest MMR globally [17].

A thorough reflection on the foregoing, in addition to the fact that no empirical study has investigated the drivers and inhibitors of SBA on a comparable ground for these countries as far as our search indicated, warranted the need for this study. This study investigated skilled birth attendance (SBA) in Sierra Leone, Niger, and Mali. Investigating the proportion of women who obtain SBA in these countries would unearth drivers and plausible inhibiting factors associated with SBA. Consequently, we explored SBA rates and associated predictors in Sierra Leone, Niger, and Mali in order to draw the attention of governments of these countries, their partner maternal health non-governmental organizations, and other developing countries to critical factors that need to the considered to accelerate SBA.

## Methods

### Data source

The study made use of pooled data from current DHS conducted in Mali (2012–2013), Niger (2012), and Sierra Leone (2013). DHS is a nationwide survey collected every 5 years across low- and middle-income countries in Africa and Asia. Women aged 15–49 years who are in their reproductive age are interviewed. For the purpose of this study, only women who had information on birth history in 5 years before the survey were included. A total of 6502 women were sampled from Mali, 7432 women from Niger, and 6461 women from Sierra Leone. The Institutional Review Board of the Inner City Fund (ICF) and Ethics Committees of the Ministries of Health in Sierra Leone, Niger, and Mali approved the surveys. Permission to use the DHS data sets was granted by MEASURE DHS. The data set is accessible to the public at https://dhsprogram.com/data/available-datasets.cfm.

### Description of variables

The main outcome variable was skilled birth attendance. The outcome variable was derived from the response to the question “Who assisted with the delivery?” Responses were categorized under health personnel and other person. Health personnel included doctor, nurse, nurse/midwife, and auxiliary midwife; Other people also consisted of traditional birth attendant (TBA), traditional health volunteer, community/village health volunteer, neighbours/ friends/relatives, other. For the purpose of the study, skilled birth attendance referred to births assisted by a doctor, nurse, auxiliary midwife, or nurse/midwife.

The explanatory variables consist of residence, age, wealth status, women and partner’s level of education, marital status, health insurance, number of antenatal care (ANC) visits, skilled ANC provider, getting medical help for self, money needed for treatment, distance to health facility and getting permission to go, listening to radio, and watching television. Residence was categorized as urban and rural. Age was grouped in 5 – year interval and captured as 15–19, 20–24, 25–29, 30–34, 35–39, 40–44, and 45–49. Wealth status was categorized as the poorest, poorer, middle, richer, and richest. Women and partner’s levels of education were captured as no education, primary, secondary, and higher education. Marital status was captured as married, cohabitation, widowed, divorced, and separated. Health insurance was categorised as yes and no. The number of antenatal care (ANC) visits was captured as less than four visits and four or more visits. Skilled ANC provider was categorised as no 0 and yes 1. Getting medical help for self, money needed for treatment, distance to health facility, and getting permission to go were captured as a big problem and not a big problem. Listening to radio was recorded as not at all, less than once a week, and at least once a week. Watching television was captured as not at all, less than once a week, and at least once a week.

### Data analysis

All analyses were done using Stata version 14. Inferential and descriptive analyses were done. Descriptive analysis was reported using tables and figures. Inferential analysis was used to examine the relationship between the explanatory variables and the outcome variable. Specifically, binary logistic regression was conducted. All results of the binary logistic analyses were presented as odds ratios (ORs), with 95% confidence intervals (CIs). The complex nature of the sampling structure of the data was adjusted using the Stata Survey command ‘svyset v021 [pweight=wt], strata (v023).’

### Ethics approval

The Institutional Review Board of ICF and Ethics Committees of the Ministries of Health in Sierra Leone, Niger, and Mali approved the surveys. Either written or verbal consent was provided by the women who participated in the surveys. We had permission to use the dataset from MEASURE DHS after our request was granted on 15th March 2019.

## Results

### Descriptive results

**Fig. 1:**
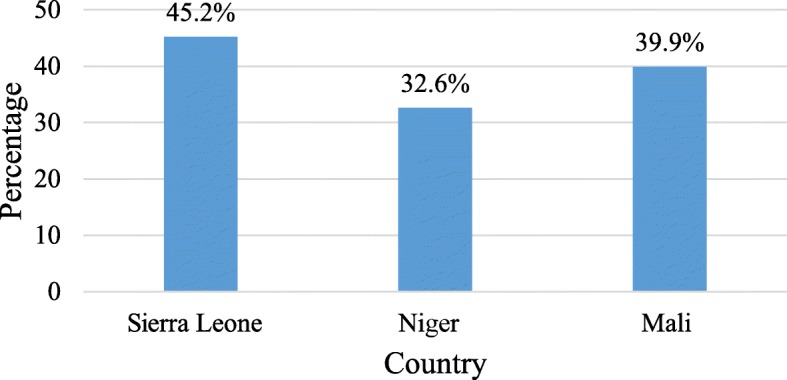
Prevalence of Skilled Birth Attendance. Source: Computed from Mali DHS (2012–2013), Niger DHS (2012) and Sierra Leone DHS (2013)

Among the three countries, SBA ranged between 32.6% (in Niger) and 45.2% (in Sierra Leone), as indicated in Fig. 1. Table 1 presents SBA with key background characteristics. Women in the 45–49 age category had the least SBA in all the three countries: Sierra Leone (34.7%), Niger (23.2%), and Mali (24.7%). In all these countries, high rates were reported by urban residents, with Mali urban women having 86.5%.

**Table 1:** Background characteristics and skilled birth attendance

Variables	Sierra Leone (*n* = 6461)		Niger (*n* = 7432)		Mali (*n* = 6502)	
	Frequency	Utilised SBAs at delivery (%)	Frequency	Utilised SBAs at delivery (%)	Frequency	Utilised SBAs at delivery (%)
Age						
15–19	417	45.5	542	35.4	534	37.9
20–24	1150	48.8	1548	34.8	1343	39.9
25–29	1677	48.3	1916	32.6	1738	40.9
30–34	1350	45.0	1558	30.9	1369	42.0
35–39	1118	42.5	1064	32.3	925	38.7
40–44	472	38.4	573	33.3	441	39.0
45–49	277	34.7	231	23.2	152	24.7
Place of residence						
Rural	1544	37.7	1042	24.2	1283	28.4
Urban	4917	69.1	6390	84.6	5219	86.5
Level of education						
No education	4638	40.2	6303	27.1	5404	34.6
Primary	938	49.3	760	56.3	585	52.5
Secondary	822	65.2	343	76.8	470	79.1
Higher	63	94.4	26	98.0	43	96.4
Wealth status						
Poorest	1534	32.9	1465	13.5	1339	16.4
Poorer	1394	36.1	1477	21.3	1360	21.7
Middle	1397	39.1	1537	25.0	1326	24.7
Richer	1226	54.6	1566	33.0	1321	55.9
Richest	910	75.9	1387	72.9	1156	87.4
Marital status						
Married	5862	44.6	7256	32.2	6360	39.6
Cohabitation	268	54.3	10	51.9	71	55.7
Widowed	111	48.6	43	32.7	28	17.7
Divorced	40	36.7	113	53.3	21	60.5
Separated	180	50.7	10	74.9	22	82.8
Partner’s educational level						
No education	4272	37.4	6072	26.5	5356	34.2
Primary	552	48.7	813	49.9	474	48.3
Secondary	1318	62.8	421	69.1	550	77.2
Higher	319	71.3	126	93.7	122	86.6
Health Insurance						
No	6421	45.0	7316	31.9	6356	39.7
Yes	40	73.6	116	80.4	146	47.3
Number of ANC visits						
Less than four	810	25.9	5000	26.8	3803	24.1
Four or more	5651	48.0	2432	44.7	2699	62.1
Skilled ANC provider						
No	2056	4.7	1285	4.4	3436	7.2
Yes	4405	64.1	6147	38.5	3066	76.5
Getting medical help for self: money needed for treatment						
Big problem	4566	40.3	4561	28.4	3445	33.7
Not a big problem	1895	57.0	2871	39.3	3057	46.8
Getting medical help for self: distance to health facility						
Big problem	2937	30.5	3278	24.4	2359	28.8
Not a big problem	3524	57.5	4154	39.1	4143	46.2
Getting medical help for self: getting permission to go						
Big problem	1117	43.5	1534	33.4	1911	36.4
Not a big problem	5344	45.6	5898	32.4	4591	41.3
Listening to radio						
Not at all	2735	33.3	2679	24.9	1969	28.8
Less than once a week	1398	52.7	2182	32.2	1535	36.6
At least once a week	2328	54.6	2571	41.1	2998	48.8
Watching television						
Not at all	5690	41.5	5795	25.0	3399	26
Less than once a week	290	64.6	826	40.6	1242	41.7
At least once a week	481	77.1	811	79.0	1861	63.7

*Source* Computed from Mali DHS (2012–2013), Niger DHS (2012) and Sierra Leone DHS (2013)

At least 9 out of 10 women with higher educational status had SBA in Sierra Leone (94.4%), Niger (98.0%), and Mali (96.4%). Falling within the richest wealth status was associated with high SBA for all the three countries: Sierra Leone (75.9%), Niger (72.9%), and Mali (87.4%). More than half of the cohabiting Sierra Leonean women had SBA (54.3%). In Niger, 7 out of 10 separated women obtained SBA (74.9%) whereas 8 out of 10 separated women in Mali had SBA (82.8%). For all these countries, the highest SBA occurred among women whose partners had higher education, and this was much phenomenal among those from Niger (93.7%).

Women who had subscribed to the National Health Insurance Scheme reported high SBA particularly in Niger (80.4%). Having four or more antenatal care (ANC) visits was consistent with relatively high SBA in Sierra Leone (48.0%), Niger (44.7%), and Mali (62.1%). Women who had the service of skilled providers during ANC reported high SBA in all countries, particularly in Mali (76.5%). Reporting that obtaining money for medical treatment was not a big problem was associated with high SBA in all the countries, especially in Sierra Leone (57.5%).

Similarly, higher SBA was reported among women who indicated that distance to the health facility was not a major challenge in all the countries, especially Sierra Leone (57.5%). Women who indicated that obtaining permission to seek healthcare was not a challenge reported higher SBA in Sierra Leone (45. 6%). SBA was highly pronounced among women who listened to radio at least once a week, especially among those from Sierra Leone (54.6%). Nearly 8 out of 10 women from Niger who watched television at least once a week had SBA (79.0%).

### Multivariate logistic regression of background characteristics and SBA

In Table 2, we present the multivariate results for background characteristics and SBA. With women aged 15–19 as the reference category, those aged 25–29 had a higher likelihood of SBA in Sierra Leone (OR 1.24; 95% CI 0.87–1.77) but all women in Niger had less SBA odds, especially those aged 45–49 (OR 0.61; 95% CI 0.36–1.03). In Mali, falling within the 40–44 age category was associated with relatively higher chances of SBA (OR 1.46; 95% CI 0.95–2.26). When checked among the three countries, women aged 45–49 had fewer odds of SBA (OR 0.66; 95% CI 0.45–0.98). Rural women had less likelihood of having SBA for all the three countries (OR 0.21; 95% CI 0.16–0.28), with the extreme occurring in Niger (OR 0.19; 95% CI 0.14–0.28).

**Table 2:** Multivariate logistic regression of background characteristics and skilled birth attendance

Explanatory Variables	Sierra Leone	Niger	Mali	All Countries
	Odds Ratio (95% CI)	Odds Ratio (95% CI)	Odds Ratio (95% CI)	Odds Ratio (95% CI)
Age				
15–19	Ref	Ref	Ref	Ref
20–24	1.23(0.86–1.77)	0.75(0.55–1.02)	1.10(0.76–1.59)	0.87(0.69–1.09)
25–29	1.24(0.87–1.77)	0.68**(0.50–0.92)	1.26(0.90–1.76)	0.89(0.72–1.11)
30–34	1.03(0.70–1.53)	0.67**(0.50–0.88)	1.18(0.84–1.68)	0.88(0.71–1.09)
35–39	1.03(0.70–1.50)	0.68**(0.51–0.93)	1.19(0.79–1.76)	0.86(0.68–1.09)
40–44	0.87(0.58–1.33)	0.80(0.56–1.14)	1.46(0.95–2.26)	0.99(0.76–1.30)
45–49	0.81(0.52–1.29)	0.61(0.36–1.03)	0.86(0.45–1.63)	0.66**(0.45–0.98)
Place of residence				
Urban	Ref	Ref	Ref	Ref
Rural	0.70(0.44–1.11)	0.19***(0.14–0.28)	0.35***(0.24–0.52)	0.21***(0.16–0.28)
Level of education				
No education	Ref	Ref	Ref	Ref
Primary	1.04(0.84–1.29)	1.74***(1.38–2.20)	0.87(0.64–1.17)	1.37**(1.14–1.63)
Secondary	1.27(0.95–1.72)	1.70**(1.14–2.54)	1.59(0.95–2.66)	1.66**(1.24–2.22)
Higher	5.41**(1.53–19.56)	3.05(0.38–24.60)	2.33(0.48–11.24)	2.50(0.72–8.69)
Health Insurance				
No	Ref	Ref	Ref	Ref
Yes	1.20(0.35–4.11)	1.48(0.75–2.91)	1.05(0.57–1.95)	1.39(0.88–2.20)
Wealth status				
Poorest	Ref	Ref	Ref	Ref
Poorer	1.04(0.81–1.34)	1.51**(1.19–1.91)	1.63**(1.21–2.17)	1.41***(1.19–1.68)
Middle	1.04(0.78–1.37)	1.65**(1.23–2.23)	1.42**(1.03–1.95)	1.41**(1.15–1.76)
Richer	1.20(0.84–1.71)	2.02***(1.52–2.67)	4.03***(2.80–5.79)	2.09***(1.67–2.61)
Richest	1.07(0.62–1.86)	2.98***(2.05–4.34)	6.21***(3.64–10.60)	2.49***(1.83–3.40)
Marital status				
Married	Ref	Ref	Ref	Ref
Cohabitation	0.83(0.60–1.15)	2.14(0.48–9.55)	0.82(0.33–2.02)	1.43(0.62–3.29)
Widowed	1.08(0.57–2.04)	1.05(0.46–2.33)	0.28*(0.09–0.94)	0.68(0.34–1.38)
Divorced	0.50(0.23–1.09)	1.61(0.87–2.97)	0.99(0.29–3.36)	1.22(0.69–2.15)
Separated	0.84(0.49–1.44)	3.37 (0.73–15.54)	1.66(0.33–8.30)	2.16(0.64–6.33)
Partner’s educational level				
No education	Ref	Ref	Ref	Ref
Primary	1.21(0.90–1.63)	1.54***(1.24–1.92)	0.87(0.33–2.02)	1.17(0.97–1.41)
Secondary	1.59***(1.28–1.99)	2.30***(1.65–3.21)	1.14(0.79–1.66)	1.78***(1.40–2.26)
Higher	1.61**(1.11–2.33)	3.48**(1.53–7.92)	0.59(0.30–1.16)	1.40(0.80–2.42)
Number of ANC visits				
Less than four	Ref	Ref	Ref	Ref
Four or more	2.18***(1.68–2.83)	1.40***(1.18–1.66)	1.60***(1.30–1.96)	1.63***(1.43–1.86)
Skilled ANC provider				
No	Ref	Ref	Ref	Ref
Yes	29.34***(21.90–39.31)	7.92***(5.59–11.2)	26.63***(21.25–33.38)	11.51***(9.53–13.92)
Getting medical help for self: money needed for treatment				
Big problem	Ref	Ref	Ref	Ref
Not a big problem	1.11(0.90–1.39)	0.95(0.80–1.12)	1.04(0.83–1.31)	1.08(0.94–1.24)
Getting medical help for self: distance to health facility				
Big problem	Ref	Ref	Ref	Ref
Not a big problem	2.07***(1.64–2.61)	1.56(0.80–1.13)	1.51**(1.18–1.94)	1.66***(1.44–1.92)
Getting medical help for self: getting permission to go				
Big problem	Ref	Ref	Ref	Ref
Not a big problem	0.72(0.52–1.02)	1.00(0.77–1.29)	0.65**(0.49–0.87)	0.69***(0.56–0.84)
Listening to radio				
Not at all	Ref	Ref	Ref	Ref
Less than once a week	1.47**(1.14–1.88)	1.07(0.87–1.32)	0.94(0.69–1.29)	0.87 (0.72–1.05)
At least once a week	1.45**(1.13–1.87)	1.12(0.88–1.43)	1.21(0.92–1.59)	1.04(0.86–2.88)
Watching television				
Not at all	Ref	Ref	Ref	Ref
Less than once a week	1.08(0.70–1.67)	1.13(0.86–1.48)	1.07(0.80–1.41)	1.53***(1.26–1.86)
At least once a week	1.58**(1.08–2.32)	2.05***(1.50–2.79)	1.21(0.92–1.59)	2.33***(1.88–2.88)

Computed from Mali DHS (2012–2013), Niger DHS (2012), Sierra Leone DHS (2013), *CI* Confidence Interval, * *p* < 0.05, ** *p* < 0.01, *** *p* < 0.001, *Ref* Reference Category

In Sierra Leone, those with higher education were 5 times more probable to obtain SBA (OR 5.41; 95% CI 1.53–19.56). Women who had subscribed to health insurance had higher odds of attaining SBA (OR 1.39; 95% CI 0.88–2.20). As compared to poorest women, richest women were noted to have higher SBA odds in all the countries (OR 2.49; 95% CI 1.83–3.40), especially in Mali (OR 6.21; 95% CI 3.64–10.60). In Mali, the widowed were less likely to have SBA (OR 0.28; 95% CI 0.09–0.94). In Niger, women whose partners had attained higher education were more probable to obtain SBA (OR 3.48; 95% CI 1.53–7.92), as compared to their counterparts whose partners do not have formal education. On the whole, women whose partners had secondary education had a high tendency of SBA (OR 1.78; 95% CI 1.40–2.26).

## Discussion

Although the efficacy of SBA in saving maternal and newborn lives is widely acknowledged [1, 19, 20], the proportion of women attaining SBA in Sierra Leone, Niger, and Mali is low, compared to other sub-Saharan African countries [21–23]. This necessitated the study to reveal the proportion of women seeking SBA and associated predictors to unearth critical factors for policy-driven SBA interventions and advocacy. Place of residence, wealth status, number of ANC visits, skilled ANC provider, and watching television were significantly related to SBA.

Niger women had the least SBA whilst the highest was recorded in Mali. Less than half of the women in the reproductive age for each of these countries had SBA in the 5 years preceding the survey. Niger is a member of several international and regional treaties such as the 1978 Alma Ata Declaration of 1978, which seeks to prioritise primary health care (PHC). In May 2002, the government also adopted the Health Policy Statement, after which the Council of Ministers enacted the Health Development Plan (HDP) 2005–2009 in pursuit of maternal and child health improvement [24]. If these initiatives have not resulted in high SBA, there is the need for a critical review or other policies and interventions that would rather improve SBA. Our finding does not vary from an observation suggesting that a woman’s lifetime risk of dying as a result of childbirth or pregnancy complications in Niger is one in seven [12].

Women of all age cohorts had less likelihood of SBA, compared to women aged 15–19, in the case of Niger. However, in Mali and Sierra Leone, a high tendency of SBA was observed among women who had advanced in age, except those aged 45–49. The observed variation across the countries may be attributable to variation in societal acceptance of childbearing at teenage (15–19 years). Teens are likely to access the service of skilled personnel if they feel appreciated and welcomed by healthcare providers and the society in which they live [25]. Women who are advanced in age might be discouraged by the attitude of healthcare providers if they had a negative experience in the past, unlike those in the 15–19 age category who might be having first deliveries and, hence, fewer chances of negative delivery experience [26]. There is consistent evidence within sub-Saharan Africa on instances where the attitude of healthcare providers dissuades women from subsequently accessing maternal healthcare services [27–29]. Some efforts have been made in Niger to enhance the uptake of SBA such as the *gratuite des soins*, an intervention introduced in the mid-2000s to offer free healthcare for maternity and under-fives [30].

A similar intervention has been instituted in Mali, where caesarean sections are offered free of charge in addition to some maternal services [30]. Our findings imply that these efforts need to be reconsidered, especially in the case of Niger. Burgess [31] noted that Nigerien women are faced with several challenges in utilising the existing maternal health system and, as such, are unable to obtain the required service, and this could partly result in the relatively less SBA, as observed in our study.

Rural residents had less likelihood of SBA, as compared to women in urban settings. Considering how healthcare facilities are skewed within these countries in favour of urban settings, it is expected for urban residents to have high SBA. For instance, Mali is one of the world’s poorest countries, with only 2.9% of its gross domestic product (GDP) invested in healthcare, and has much healthcare concentration in its capital, Bamako [32]. Bamako alone has over 4030 (55%) healthcare providers whereas 3279 (45%) healthcare providers cater to the health needs of the residents outside the capital, indicating that women in remotest regions would struggle to attain SBA [33].

Similarly, in Sierra Leone, Kingham et al. [34] observed that 90% of surgeons are confined to Freetown, the capital. Data from the Human Resources for Health revealed that between 2005 and 2011, the doctor-patient ratio in Sierra Leone increased from 0.07 to 0.12 per 1000 in the western sector where the capital is located. The corresponding increase in the rural sector (Koinadugu) was from 0.03 to 0.05 per 1000 population, and the nurse-patient ratio reflected the same [35]. The post-conflict fragility of the healthcare system in Sierra Leone [36] could partly account for the rural-urban disparity in SBA. The rural-urban disparity is not only peculiar to these countries but resonates well with literature from both developed and developing countries [37–39].

We found that the higher a woman or her partner’s educational status, the higher the likelihood of SBA, as compared to women who neither have formal education nor their partners, and this was consistent for all the three countries except in Mali. Generally, educated women become more knowledgeable and conscious of their health, have more antenatal care visits, and eventually desire to obtain SBA. This outcome is consistent with some evidence from Kenya, Ethiopia, and other developing countries [40–42].

We observed that, for all the three countries, the higher a woman’s wealth standing, the higher the likelihood for SBA. Having what it takes economically to gratify one’s needs is a prerequisite to satiate that particular need. There might be National Health Insurance Scheme (NHIS), yet transportation costs can hinder a woman from accessing SBA. However, this cannot deter a wealthier woman from accessing skilled birth because she has the financial means and can even arrange for home delivery. The fact that wealthier women have a higher inclination toward SBA in Sierra Leone, Mali, and Niger, as observed in the present study, has consistently been reported in the literature from a number of low- and middle-income countries [39, 43–45].

In this present study, separated and divorced women had a high likelihood of SBA, as compared to married women. Could this imply their relative autonomy/empowerment as compared to women with partners? Inconsistent findings have been reported. In Ghana, Ameyaw et al. [46] indicated that married women had higher SBA inclination, compared to women who were not in any marital union. Afulani and Moyer [40], however, noted a higher likelihood of SBA among women who had never married and those who had previously married, as compared to women who were currently married.

Women who had four or more ANC visits and having skilled ANC providers were associated with a high likelihood of SBA. Similar to our findings, a Zambia-based study has also illustrated the positive association between ANC and SBA [47]. ANC is expected to be a precursor to delivery for all women globally and due to its proven gains in safeguarding maternal and newborn health, the latest WHO recommendations require women from developing world such as Mali, Sierra, and Niger to have a minimum of eight (8) ANC visits [48].

Women who reported that distance to health facilities was not a major challenge had a higher likelihood of SBA. Similarly, those who were either listening to radio or watching television at least once a week had a higher likelihood of SBA. This finding can be linked to the reproductive health initiatives that utilised the media. For instance, through the Strengthening Reproductive Health Project (SRHP), television and radio emissions were used to influence the reproductive health behaviour of about 14 million people in Mali [49]. Gaining the right information from the media (radio/television) and having no difficulty in accessing a lifesaving service are enablers for utilising that particular service. These findings coincide with some prior studies that also inquired maternal health utilisation [39, 44, 50].

### Strength and limitations

The use of large national-level comparable surveys (DHS) enhances the generalisability of our findings to other developing countries, which is a major strength of this study. However, the results need to be interpreted cautiously, as a cross-sectional study design does not permit causal-effect attribution of the observation made.

## Conclusion

The study has revealed that less than half of women in Mali, Sierra Leone, and Niger utilise SBA. Having ANC with a skilled provider, being rich, and living in an urban location are essential conditions for SBA. Efforts to increase the current SBA rates in these countries ought to focus on the rural-urban disparity in healthcare, female education, and ANC attendance among these countries. The need to review existing policies, interventions, and programs aimed at improving maternal health conditions is critical, especially in Niger. Other pro-poor interventions might be needful considering the multidimensional nature of poverty. Incentives to yield equitable distribution of healthcare providers, especially among midwives and physicians, could as well be of relevance in attempts to make SBA handier for all women irrespective of location. These recommendations could apply to other sub-Saharan African countries struggling to elevate their current SBA rates.

## Data Availability

The datasets supporting the conclusions of this article are available in the Measure DHS repository, https://dhsprogram.com/data/available-datasets.cfm.

## Abbreviations

ANC: Antenatal Care
AOR: Adjusted Odds Ratio
CI: Confidence Interval
DHS: Demographic and Health Survey
FHCI: Free Health Care Initiative
GDP: Gross Domestic Product
HDP: Health Development Plan
ICF: Inner City Fund
MCSP: Maternal and Child Survival Program
MMR: Maternal Mortality Ratio
NHIS: National Health Insurance Scheme
OR: Odds Ratio
PHC: Primary Health Care
SBA: Skilled Birth Attendant
SDG: Sustainable Development Goal
TBA: Traditional Birth Attendant
UNICEF: United Nations Children’s Fund
USAID: United States Agency for International Development
WHO: World Health Organisation
